# The impact of generative artificial intelligence (GenAI) usage patterns on employee creativity

**DOI:** 10.3389/fpsyg.2026.1827568

**Published:** 2026-05-11

**Authors:** Qiang Li, Shuangshuang Li

**Affiliations:** School of Business Administration, Lanzhou University of Finance and Economics, Lanzhou, China

**Keywords:** employee creativity, generative artificial intelligence (GenAI), knowledge integration capability, self-determination theory, task complexity

## Abstract

**Introduction:**

Generative AI (GenAI) is transforming the way employees innovate, yet existing research has mainly focused on its adoption contexts and usage frequency.

**Methods:**

Based on a three-wave lagged survey design with 381 valid matched employees from knowledge-intensive firms in China, this exploratory empirical study develops and tests a model grounded in the exploration–exploitation framework, with knowledge integration capability as the core mediator and task complexity as the moderator.

**Results:**

Results show that: (1) Exploitative GenAI use is more strongly positively associated with incremental creativity than radical creativity; (2) Exploratory GenAI use is more strongly positively associated with radical creativity than incremental creativity; (3) Employees’ knowledge integration capability plays a critical complementary mediating role in the above relationships; (4) Task complexity positively moderates the relationships between GenAI usage patterns and knowledge integration capability.

**Discussion:**

This study improves the theoretical precision of understanding human-AI co-creativity and provides evidence-based guidance for organizational GenAI strategies.

## 1 Introduction

Generative artificial intelligence (GenAI) has ushered in a new era of artificial intelligence research and application characterized by intelligent content creation (Burger et al., 2023). Unlike earlier AI systems, GenAI can autonomously produce high-fidelity multimodal outputs, including text, code, images, and audio (Das and Varshney, 2022), thereby demonstrating unprecedented creative capabilities. Its adoption has expanded rapidly across industries, both in breadth and depth, with the number of generative AI users in China reaching 515 million as of June 2025.^1^ As a core technological driver of the digital age, GenAI is fundamentally reshaping organizational work practices, mechanisms of knowledge creation, and paradigms of innovation development (Wilson and Daugherty, 2018). Consequently, developing responsible and evidence-based strategies for GenAI integration has become a strategic imperative for organizations seeking to enhance innovation and improve performance. At the same time, however, excessive reliance on GenAI may foster homogenized thinking and diminish creative diversity, thereby constraining radical innovation.

Accordingly, GenAI is widely regarded in the management literature as a double-edged sword. On the one hand, it can augment human cognitive capacities, empower employees in their daily work, and stimulate creative thinking in problem solving (Shao et al., 2022). On the other hand, emerging empirical evidence has highlighted several significant risks associated with its use, including employees’ cognitive dependence on the technology, task displacement driven by intelligent automation, and the erosion of human agency in the workplace. These concerns have raised urgent questions regarding the quality, sustainability, and equity of human–AI collaboration in organizational settings (Verma and Vibhav, 2022). Precisely identifying the psychological, behavioral, and contextual mechanisms through which GenAI influences employee innovation, as well as the boundary conditions that shape these effects, is therefore of considerable theoretical importance and practical urgency.

Existing research on GenAI and employee innovation has primarily focused on the antecedents of GenAI adoption and surface-level indicators of use. Prior studies have examined factors such as individuals’ intentions to adopt GenAI (Mao et al., 2024), domain-specific contexts of application (You, 2023), and the frequency or intensity of GenAI use (Noy and Zhang, 2023). Yet a substantial gap remains in understanding how qualitatively distinct patterns of GenAI use shape employees’ innovative behaviors and outcomes. Such patterns may include purpose-driven versus habitual engagement, collaborative co-creation with GenAI versus autonomous delegation of tasks to it, and reflective versus reactive interaction with GenAI systems. Moving beyond the binary question of whether employees should use GenAI, the present study adopts a process-oriented perspective to examine how employees use GenAI and how different usage patterns mediate or moderate its effects on innovation.

Drawing on exploration–exploitation framework (March, 1991) as the core theoretical foundation, with self-determination theory (Ryan and Deci, 2000) as supportive motivational background, this study develops and empirically tests a moderated mediation model to explain how exploitative and exploratory patterns of GenAI use differentially influence employees’ incremental and radical creativity. By doing so, it offers empirically grounded and actionable insights for managers seeking to align GenAI training, tool design, and implementation practices with employees’ job roles, organizational innovation goals, and the specific demands of work contexts. Overall, this study adopts an exploratory perspective to investigate how different patterns of GenAI use are associated with employees’ creativity.

Notably, this study offers clear theoretical and empirical contributions that complement and meaningfully extend the insightful work of Zhang et al. (2025) on GenAI use and differentiated employee creativity. Four key distinctions establish our research as a valuable incremental addition to the literature. First, while Zhang et al. (2025) classify GenAI use by purpose, distinguishing between work-related and nonwork-related application, we focus on employees’ cognitive orientation by separating exploitative use from exploratory use. This approach highlights how employees engage with GenAI at the cognitive level, rather than only where or why GenAI is applied. Second, Zhang et al. (2025) propose exploratory and exploitative learning as mediating variables, whereas our study identifies knowledge integration capability as the core cognitive mediator, which helps clarify the underlying black box between GenAI use and creativity. Third, departing from the focus on perceived ease of use in Zhang et al. (2025), we examine task complexity as a moderator, which carries greater relevance for innovation in knowledge-intensive work. Finally, we employ a three-wave time-lagged design to strengthen temporal rigor and reduce common method bias, while Zhang et al. (2025) rely on cross-sectional data. Collectively, these distinct features demonstrate the unique, complementary, and theoretically grounded contribution of this study.

## 2 Literature review

### 2.1 Generative artificial intelligence (GenAI)

Generative Artificial Intelligence (GenAI) refers to computational systems capable of producing multimodal outputs, including text, images, audio, and video, by leveraging large-scale pre-trained models and extensive datasets. Beyond its robust content-generation capabilities, GenAI exhibits a notable degree of flexibility and contextual adaptability, approaching certain hallmarks of human-like intelligence (Taeihagh, 2025). This technology is diffusing across industries at an unprecedented rate, fundamentally transforming conventional work structures and professional practices. In light of this rapid integration, an expanding body of scholarly inquiry has turned its attention to critical implications of GenAI adoption, particularly concerning work efficiency, task redesign, and the evolving dynamics of human-AI collaboration (Acemoglu and Restrepo, 2018). Nevertheless, substantial conceptual and empirical gaps persist in both academic understanding and practical implementation of GenAI, underscoring the need for more nuanced, theory-informed investigation.

First, prevailing research largely operationalizes “GenAI use” as a unidimensional and homogeneous construct—focusing predominantly on linear associations between usage frequency, self-reported willingness to adopt, and downstream performance metrics (Budhwar et al., 2023). Yet the relationship between GenAI and employee innovation is neither uniform nor linearly facilitative. Emerging evidence points to an “innovation paradox”: while GenAI can serve as a potent catalyst for ideation—enabling rapid exploration of novel concepts and accelerating breakthrough idea generation (Holmström and Carroll, 2025; Guo, 2026)—excessive or uncritical reliance on such systems may erode employees’ cognitive engagement, weaken analytical rigor, and foster convergent, homogenized thinking patterns. Consequently, autonomous reasoning, original insight formation, and creative problem-solving capacities may be inadvertently constrained (Yin et al., 2024; Wang and Wang, 2026; Wang et al., 2026). This tension implies that GenAI’s influence on innovation is contingent not on “whether” it is used, but rather on “how,” “for what purposes”, and “under what conditions” it is integrated into cognitive and collaborative workflows.

Employee engagement with GenAI is inherently heterogeneous and purpose-driven. Some individuals primarily deploy GenAI to automate routine tasks or retrieve context-specific solutions (Zhang et al., 2025), thereby validating, refining, and adapting existing workflows and knowledge artifacts to meet localized operational needs. Others leverage GenAI to explore unfamiliar domains, acquire novel competencies, engage in iterative trial-and-error experimentation, integrate knowledge across disciplinary boundaries, and generate unconventional solutions. Although the exploration-exploitation framework was initially proposed to explain organizational learning behavior (March, 1991), existing studies have validated its applicability to the individual level (Mom et al., 2007; Zheng et al., 2023), as individual work behavior is the micro-foundation of organizational learning and innovation. In the context of GenAI usage, exploitative and exploratory use at the individual level reflects employees’ different cognitive processing styles and knowledge acquisition goals, which is consistent with the core logic of the exploration-exploitation framework and thus applicable to this study. These two distinct usage patterns may differentially influence employees’ innovative behavior. Consequently, understanding “how” GenAI should be deployed—rather than merely “whether” it is adopted—to more effectively stimulate employee innovation constitutes a timely and theoretically significant question for scholarly inquiry.

### 2.2 Self-determination theory (SDT)

Self-Determination Theory (SDT) serves as a supportive motivational background rather than the core theoretical framework of this study. It provides a general explanation for why different usage patterns can motivate cognitive engagement and knowledge processing.

Given its supportive explanatory power regarding motivational processes, SDT has been increasingly applied in organizational research examining how GenAI, shapes employee behavior. For instance, Zhang et al. (2025) leveraged SDT to investigate the mediating role of employee learning behaviors in the relationship between GenAI usage and creativity. Their findings reveal that GenAI use enhances learning engagement, which subsequently strengthens creative performance. Similarly, Kong et al. (2024) demonstrated that employees’ perception of AI as supportive—particularly in enabling choice, mastery, and interpersonal connection—satisfies their need for autonomy, thereby encouraging proactive exploration and ultimately improving innovative performance among hospitality professionals. Collectively, these studies underscore SDT’s conceptual utility and empirical relevance for investigating how GenAI integration can foster employee innovation.

This reallocation of cognitive capacity constitutes a critical precondition for activating knowledge integration capability: the ability to synthesize, recombine, and reconfigure existing and newly acquired knowledge across disciplinary and functional boundaries (Jian et al., 2022). Such integrative capacity, in turn, serves as a foundational enabler of innovative behavior.

To further delineate the boundary conditions of this mechanism, the present study introduces task complexity as a moderating variable. Prior research on how enterprise digital technologies enhance employees’ improvisational capabilities has identified task complexity as a key contingency factor shaping the effectiveness of technology adoption (Chan et al., 2015). Specifically, the empowering effect of technology on employee cognition and capability development is more pronounced under conditions of high task complexity (Leonardi, 2014). When confronted with highly complex tasks, employees experience heightened demands for external knowledge and novel, adaptive solutions. In such contexts, the value of GenAI as a potent cognitive augmentation tool becomes especially salient. Exploitative use of GenAI—characterized by its application to refine, optimize, and extend existing knowledge and practices—more effectively alleviates the cognitive load imposed by complex tasks, thereby strengthening employees’ sense of competence and facilitating integrative knowledge application. Likewise, exploratory use—oriented toward discovery, experimentation, and the synthesis of disparate knowledge domains—more robustly satisfies employees’ need for autonomy and catalyzes radical knowledge integration when task complexity is high. In contrast, for routine or low-complexity tasks, employees’ reliance on and perceived need for GenAI diminish, attenuating the proposed psychological and cognitive empowerment pathways. Consequently, incorporating task complexity enables a more precise theoretical specification of the conditions under which GenAI empowers employee innovation—thereby advancing scholarly understanding of the practical managerial question: “Under what conditions is this effect most pronounced?”

## 3 Theoretical foundation and hypothesis development

In summary, this study proposes that exploitative and exploratory uses of GenAI enhance employees’ innovative capabilities by providing differentiated cognitive inputs and knowledge resources, thereby fostering knowledge integration. Knowledge integration capability—the cognitive process of synthesizing, recombining, and reconfiguring knowledge—is thus most effectively activated when employees operate within the synergistic positive psychological states of competence, autonomy, and relatedness. This activation, in turn, naturally augments their innovative capacity. Notably, task complexity moderates the extent to which distinct GenAI usage patterns influence employees’ innovative outcomes.

### 3.1 GenAI usage and employee knowledge integration capability

Distinct from prior technologies, GenAI can perform human-like cognitive tasks—such as reasoning and problem-solving (Dennis Alan et al., 2023)—and is therefore increasingly conceptualized as a “cognitive partner” that enables deep, synergistic collaboration with employees and enhances organizational creativity (Seeber et al., 2020). While extant research on GenAI has evolved from initial investigations of adoption drivers (Pillai et al., 2024) to more nuanced examinations of post-adoption usage patterns (Zhang et al., 2025), a critical gap persists: the purpose-driven nature of GenAI usage—and its differential impact on individual and organizational outcomes—remains largely underexplored. To address this gap, the present study introduces a theoretically grounded distinction rooted in fundamental motivational orientations: exploitative use vs. exploratory use. Exploitative use refers to the application of GenAI to verify, refine, and select optimal solutions from existing work outputs and established knowledge bases (March, 1991). In contrast, exploratory use denotes the deployment of GenAI to acquire novel knowledge, probe uncharted possibilities, and generate original ideas—such as proactively learning new competencies or venturing into emerging domains (Zhang et al., 2024). This dual-purpose framework provides a vital analytical lens for disentangling the distinct mechanisms through which GenAI influences employee innovation.

Knowledge integration capability is defined as an individual’s or organization’s ability to identify, assimilate, transform, and systematically reconfigure dispersed and heterogeneous knowledge to generate novel knowledge or solutions (Grant, 1996). When employees adopt an “exploitative” usage pattern, GenAI helps them organize and optimize existing knowledge, thereby promoting the integration and reorganization of current professional knowledge. In contrast, exploratory GenAI use provides cross-domain and heterogeneous knowledge inputs, encouraging employees to connect new knowledge with existing expertise and build integrative cognitive networks. Based on this reasoning, we hypothesize:

H1a: Exploitative use of GenAI is positively related to employees’ knowledge integration capability.

H1b: Exploratory use of GenAI is positively related to employees’ knowledge integration capability.

### 3.2 Knowledge integration capability and employee creativity

Employee innovative behavior, also referred to as employee creativity or employee innovative capability in existing literature (Tang et al., 2022; Ren and Song, 2024), reflects the process and outcome of employees generating and implementing novel and valuable work-related ideas, and is operationalized as a two-dimensional construct including incremental innovation and radical innovation in this study. Although prior research has largely emphasized how generative artificial intelligence (GenAI) usage enhances overall employee innovativeness, comparatively little attention has been devoted to how such usage specifically fosters distinct forms of employee creativity. Employee creativity is a multifaceted construct, encompassing two primary dimensions: incremental innovation—which entails small-scale adjustments and refinements to existing solutions to improve efficiency, effectiveness, or contextual applicability—and radical innovation—which challenges prevailing paradigms to introduce fundamentally new concepts capable of yielding transformative outcomes (Ren and Song, 2024).

Effective integration of dispersed knowledge within teams constitutes a critical mechanism for achieving superior performance and enabling creative problem solving; conversely, teams whose members fail to integrate knowledge cohesively face severe constraints on their collective creative potential (Tiwana and McLean, 2005). At the individual level, an employee’s capacity to integrate diverse knowledge sources emerges as a foundational antecedent of both enhanced knowledge utilization and heightened innovative capability (Ancona and Caldwell, 1992).

From a knowledge integration perspective, strong integration capability helps employees refine existing workflows and solutions, driving incremental creativity; meanwhile, it supports cross-boundary knowledge recombination, facilitating radical creativity. Accordingly, we propose the following hypotheses:

H2a: Employees’ knowledge integration capability has a significant positive effect on their incremental creativity.

H2b: Employees’ knowledge integration capability has a significant positive effect on their radical creativity.

### 3.3 The mediating role of knowledge integration capability

From the exploration-exploitation and knowledge integration perspective, knowledge integration—a higher-order cognitive process characterized by the synthesis and structural reorganization of fragmented, heterogeneous information with preexisting knowledge structures—constitutes a foundational antecedent of novel idea generation (Ding and Wu, 2020; Grant, 1996). We propose that differential usage patterns of GenAI foster distinct modes of knowledge integration precisely through their capacity to fulfill employees’ heterogeneous psychological needs, thereby cultivating divergent types of innovative capability.

Specifically, exploitative GenAI use focuses on optimizing existing solutions, which drives knowledge integration toward refining current practices and ultimately promotes incremental creativity. In contrast, exploratory GenAI use focuses on acquiring new knowledge and exploring unknown fields, which drives cross-boundary knowledge integration and primarily catalyzes radical creativity.

Based on these theoretical mechanisms, we propose the following hypothesis:

H3: Knowledge integration capability mediates the relationship between GenAI usage and employee creativity.

H3a: Knowledge integration capability mediates the relationship between exploitative GenAI use and incremental creativity.

H3b: Knowledge integration capability mediates the relationship between exploitative GenAI use and radical creativity.

H3c: Knowledge integration capability mediates the relationship between exploratory GenAI use and incremental creativity.

H3d: Knowledge integration capability mediates the relationship between exploratory GenAI use and radical creativity.

### 3.4 The moderating role of task complexity

Task complexity serves as a pivotal contextual moderator that shapes both the intensity of employees’ psychological needs and the strategic allocation of their cognitive resources. Based on Wood (1986) foundational conceptualization, high task complexity is defined by goal ambiguity, multiplicity of viable solution paths, and substantial information-processing demands—conditions that collectively strain employees’ cognitive capacity and adaptive flexibility. Under such conditions, the need for effective external cognitive scaffolding becomes especially acute. GenAI, functioning as a scalable and responsive cognitive partner, exhibits heightened functional value and perceived efficacy, thereby intensifying the extent to which distinct usage patterns drive strengthen knowledge integration.

Specifically, under high task complexity, employees face greater knowledge gaps and cognitive loads. Exploitative GenAI use provides targeted solutions that help employees organize and integrate existing knowledge more efficiently. By contrast, in low-complexity tasks—where goals are clear, solution paths are routine, and information demands are minimal—employees’ preexisting knowledge reserves typically suffice. Consequently, reliance on GenAI remains marginal, and the downstream catalytic effect of exploitative use on knowledge integration is attenuated. Accordingly, we hypothesize:

H4a: Task complexity positively moderates the relationship between exploitative use of GenAI and knowledge integration; specifically, the positive effect of exploitative use on knowledge integration strengthens as task complexity increases.

Conversely, high-complexity tasks require diverse and novel knowledge inputs. Exploratory GenAI use provides cross-domain knowledge, which strongly promotes knowledge integration and creative recombination. In low-complexity settings, however, the absence of cognitive friction diminishes both the necessity and pull of exploration, constraining the capacity of exploratory use to stimulate transformative knowledge integration. Thus, we propose:

H4b: Task complexity positively moderates the relationship between exploratory use of GenAI and knowledge integration; specifically, the positive effect of exploratory use on knowledge integration strengthens as task complexity increases.

**Figure 1:**
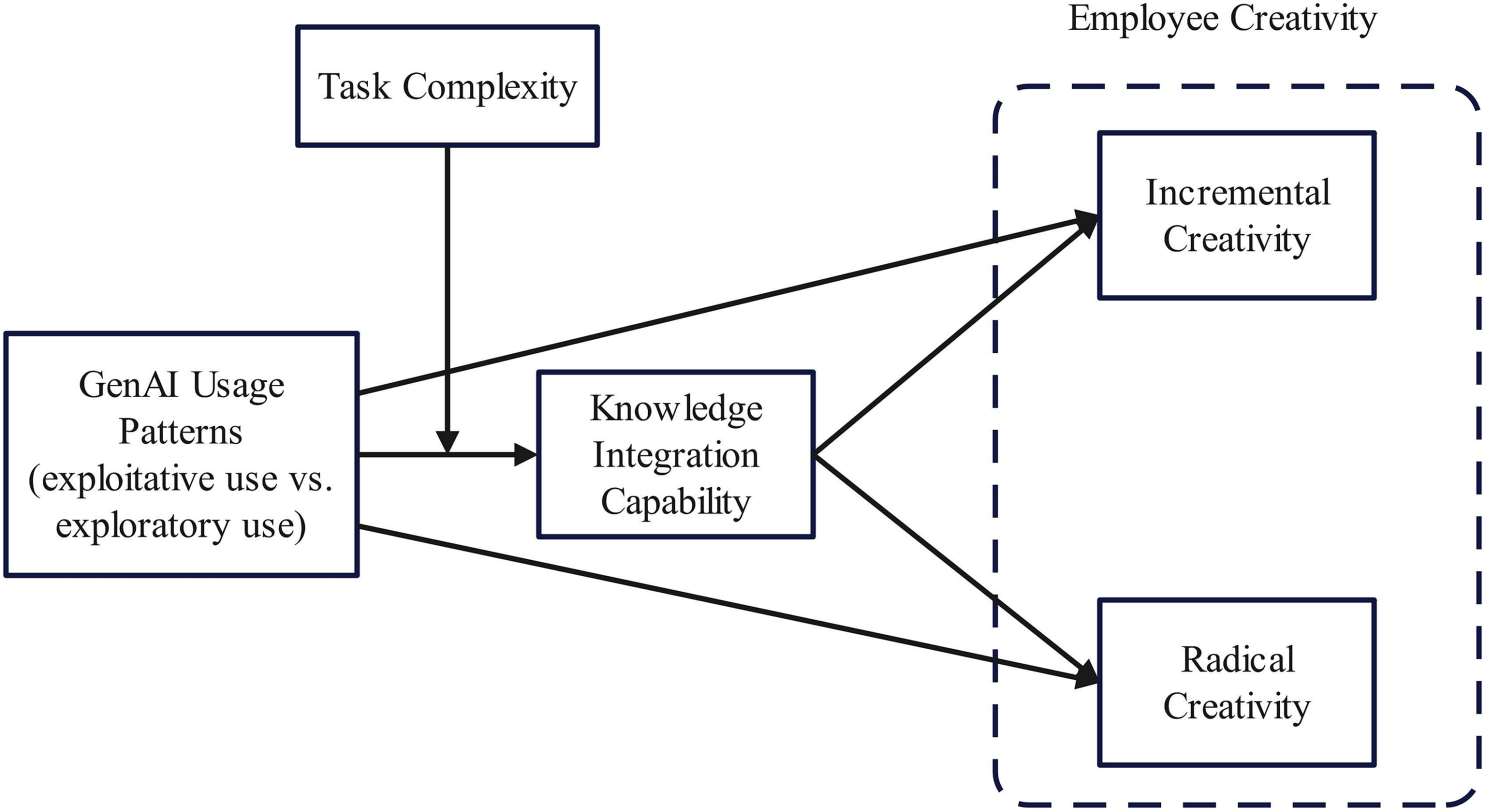
Theoretical model. Exploitative GenAI use is positively promotes knowledge integration and incremental creativity. Exploratory GenAI use is positively promotes knowledge integration and radical creativity.

In summary, integrated with theories of knowledge recombination and cognitive scaffolding, the theoretical model advanced in this study is depicted in Figure 1.

## 4 Research design

### 4.1 Sample selection and data collection

This study adopted a multi-wave questionnaire survey design to collect primary data, which mitigates the potential for common method bias (CMB) and causal ambiguity inherent in cross-sectional research (Podsakoff et al., 2003). The survey targeted full-time employees working in knowledge-intensive enterprises across high-tech, digital services, creative industries, and professional consulting sectors in China—contexts where GenAI is increasingly integrated into daily work practices and knowledge-based innovation is a core organizational objective. This sampling frame was selected based on two theoretical rationales: First, employees in knowledge-intensive firms rely heavily on cognitive processing, knowledge recombination, and creative problem-solving (Acemoglu and Restrepo, 2018; Leonardi, 2014), which are the core cognitive mechanisms linking GenAI usage to employee creativity in this study (i.e., knowledge integration capability). Second, GenAI’s core value lies in augmenting human cognitive capabilities and facilitating knowledge creation (Shao et al., 2022; Das and Varshney, 2022), and knowledge-intensive industries are the primary application scenarios where GenAI’s cognitive augmentation effect is most prominent. Thus, employees in these industries are ideal informants for examining the relationship between GenAI usage patterns and innovative behavior.

Prior to the formal survey, a pilot test was conducted with 60 employees from three knowledge-intensive enterprises (20 from each sector) to refine the measurement scales. The pilot sample was excluded from the formal study to avoid response bias. Based on the pilot test results, minor revisions were made to the wording of several scale items to enhance clarity, readability, and contextual fit (e.g., simplifying overly academic phrasing to align with employees’ daily work language). The final questionnaire comprised two sections: (1) demographic information (age, gender, education level, job tenure, position level, and industry sector) and (2) core construct measures (GenAI exploitative/exploratory use, knowledge integration capability, task complexity, and incremental/radical innovation). A brief introductory section was included to explain the study’s academic purpose, guarantee the anonymity and confidentiality of all responses, and define key terms (e.g., GenAI, incremental innovation, radical innovation) to ensure a consistent understanding among respondents.

To further reduce CMB and temporal ambiguity, the formal survey was administered in three waves with a 15-day time lag between each wave, and respondents were tracked using a unique anonymous code (combining birth year and a self-assigned 3-digit number) to match responses across waves (Podsakoff et al., 2012). The specific measurement timing for each construct was theoretically driven:

Wave 1 (T1): Measured the independent variables (GenAI exploitative use and exploratory use) and control variables (demographic characteristics), with 450 questionnaires distributed via an online professional survey platform (Wenjuanxing).

Wave 2 (T2, 15 days after T1): Measured the mediating variable (knowledge integration capability) and moderating variable (task complexity), targeting the 450 respondents from T1; 412 valid responses were collected at this stage.

Wave 3 (T3, 15 days after T2): Measured the dependent variables (incremental innovation and radical innovation), targeting the 412 respondents from T2.

After data collection, rigorous screening criteria were applied to eliminate invalid responses: (1) incomplete questionnaires (missing more than 10% of items); (2) response bias (e.g., straight-lining, identical ratings for all items); and (3) inconsistent anonymous codes across waves. A final sample of 381 valid matched questionnaires was retained, resulting in an overall valid response rate of 84.67%. This sample size meets the minimum requirements for partial least squares structural equation modeling (PLS-SEM)—Hair et al. (2014) recommend a sample size of at least 10 times the number of indicator items for the most complex construct (this study’s maximum indicator number is 4, corresponding to a minimum sample size of 40) or 300 for general PLS-SEM analysis, confirming the adequacy of the sample for subsequent hypothesis testing.

To improve response rates and reduce non-response bias, a small monetary incentive (¥1–¥10, random allocation) was provided to all respondents who completed all three waves of the survey. Non-response bias was assessed by comparing the demographic characteristics (age, gender, education level, job tenure) of early respondents (first 50% of completed surveys) and late respondents (last 50%) using independent-samples t-tests (Armstrong and Overton, 1977). No significant differences were found between the two groups (all *p* > 0.05), indicating that non-response bias is not a significant issue in this study.

### 4.2 Variable measurement

All scales were adapted from psychometrically validated, high-impact studies in top management, information systems, and marketing journals, ensuring content validity and construct consistency (Hair et al., 2014). Given the Chinese context, scales underwent a rigorous back-translation procedure (Brislin, 1970): two bilingual scholars translated English scales to Chinese; a third blind back-translated to English; discrepancies were resolved via iterative discussion to ensure semantic equivalence. Minor revisions were made based on pilot test feedback to align with GenAI workplace scenarios in China.

All latent constructs were measured using a 7-point Likert scale (1 = Strongly Disagree, 7 = Strongly Agree), widely adopted for capturing nuanced perceptual and behavioral responses in organizational innovation and AI research (Zhang et al., 2025; Yin et al., 2024). Scale items and standardized factor loadings are presented in Table 1.

**Table 1:** Full collinearity assessment (VIF values).

Variable	VIF values
EUI	2.075
EUR	2.076
KIC	2.636
IC	2.259
TC	1.300
RC	2.691

#### 4.2.1 Independent variables

GenAI usage patterns (two-dimensional):

Exploitative GenAI use (EUI): 4-item scale adapted from Mom et al. (2007), measuring GenAI application to refine existing knowledge, workflows, and solutions. Sample item: “I use GenAI assistants to continuously improve existing workflows to enhance efficiency”.

Exploratory GenAI use (EUR): 4-item scale adapted from Zheng et al. (2023), measuring GenAI deployment to acquire novel knowledge and explore uncharted work possibilities. Sample item: “I use GenAI assistants to proactively explore new knowledge in certain domains”.

#### 4.2.2 Mediating variable

Knowledge integration capability (KIC): 4-item scale adapted from Tiwana and McLean (2005), operationalized at the individual level to reflect the ability to synthesize heterogeneous knowledge for novel solutions. Sample item: “I can draw knowledge from multiple specialized fields to form new work concepts”.

#### 4.2.3 Moderating variable

Task complexity (TC): 4-item scale adapted from Gupta et al. (2013), measuring subjective perceptions of task demands (goal ambiguity, information-processing requirements). Sample item: “I consider my tasks to be mentally demanding”.

#### 4.2.4 Dependent variables

Employee innovative behavior (two-dimensional):

Incremental Creativity (IC): 3-item scale adapted from Madjar et al. (2011), capturing iterative improvements to existing work procedures/ideas. Sample item: “I can easily modify pre-existing work procedures to fit current needs”.

Radical Creativity (RC): 3-item scale adapted from Madjar et al. (2011), assessing transformative, paradigm-challenging ideas. Sample item: “I propose entirely new ways of doing my work”.

#### 4.2.5 Control variables

Five variables were controlled to rule out alternative explanations: gender (1 = male, 2 = female), age (continuous, years), education level (ordinal: 1 = associate degree, 2 = bachelor’s, 3 = master’s or above), job tenure (continuous, years in current organization), and position level (ordinal: 1 = non-managerial staff, 2 = front-line supervisor, 3 = middle manager, 4 = senior management). These variables are consistently linked to technology adoption and innovative performance in prior research (Madjar et al., 2011; Pillai et al., 2024).

### 4.3 Analytical methods

Structural Equation Modeling (SEM) was employed to test hypotheses and validate the theoretical model. Two primary SEM estimation methods were compared: Covariance-Based SEM (CB-SEM) and Partial Least Squares SEM (PLS-SEM). PLS-SEM was selected for three key reasons (Hair et al., 2011): (1) it does not require strict multivariate normality assumptions, which is suitable for self-reported data; (2) it yields robust estimates for complex models (e.g., moderated mediation) with moderate sample sizes; (3) it simultaneously assesses measurement and structural models, aligning with the study’s composite construct focus.

Data analysis was performed using SmartPLS 4 software. A Bootstrap sampling method with 5,000 resamples was used to test the significance of path coefficients. Model fit and predictive power were evaluated via R^2^ (explanatory power), Q^2^ (predictive relevance), and f^2^ (effect size) (Hair et al., 2014). Reliability and validity were assessed using Cronbach’s *α*, Composite Reliability (CR), Average Variance Extracted (AVE), confirmatory factor analysis (CFA), and Heterotrait-Monotrait Ratio (HTMT) (Fornell and Larcker, 1981; Henseler et al., 2015). Multicollinearity was checked via Variance Inflation Factor (VIF) values (Diamantopoulos, 2006).

## 5 Data analysis and hypothesis testing

### 5.1 Assessment of data quality

#### 5.1.1 Sample descriptive statistics

As shown in Table 2, the demographic characteristics of the sample are as follows. Regarding gender, male respondents accounted for 55.4%, and female respondents accounted for 44.6%, indicating a balanced gender distribution. In terms of age, the largest proportion (47.5%) fell within the 26–35 years old range. Educational attainment covered levels from associate degrees to bachelor’s degrees and master’s degrees or higher, with bachelor’s degrees or higher constituting 97.4% of the sample. Work experience was primarily concentrated in the 1–5 years range, accounting for 47.0% of respondents. In terms of employee type, the majority were non-managerial staff.

**Table 2:** Demographic information for sample.

Variables	Number	Percentage
Gender		
Male	211	55.4%
Female	170	44.6%
Age		
<25 years old	177	46.5%
26–35 years old	181	47.5%
36–45 years old	22	5.8%
>46 years old	1	0.2%
Education		
High school or below	0	0%
Associate degree	10	2.6%
Bachelor’s degree	251	65.9%
Master’s degree or above	120	31.5%
Job tenure		
<1 year	152	39.9%
1–5 years	179	47.0%
6–10 years	40	10.5%
>11 years	10	2.6%
Position level		
Non-managerial staff	296	77.7%
Front-line supervisor	57	15.0%
Middle manager	26	6.8%
Senior management	2	0.5%

#### 5.1.2 Common method Bias test

Although the data for this study were collected in three separate survey waves to mitigate potential issues, the primary data still originated from employee self-reported questionnaires. Therefore, common method bias (CMB) remains a potential concern. To ensure the reliability of the research results, this study employed two complementary post-hoc statistical methods to test for CMB. First, we used the full collinearity test proposed by Kock (2015). This method examines the variance inflation factor (VIF) for each latent variable; VIF values below 3.3 indicate that severe CMB is not a concern. The analysis showed that all VIF values in this study ranged from 1.300 to 2.691 (as shown in Table 1), which are well below the recommended threshold of 3.3 (Diamantopoulos, 2006), suggesting the absence of serious CMB. Second, we applied the Harman single-factor test (Harman, 1976). In this test, all measurement items were loaded into an exploratory factor analysis (EFA) without rotation. The analysis results indicated that the maximum single factor accounted for 39.739% of the total variance explained, which is below the recommended threshold of 50%. Furthermore, the unrotated solution extracted four factors with eigenvalues greater than one. These results suggest that common method bias is not a severe issue in this survey and is unlikely to substantially interfere with the analytical relationships among the variables (Zhou and Long, 2004).

#### 5.1.3 Tests of reliability and validity

This study primarily employed the internal consistency reliability indicator, Cronbach’s *α* coefficient, and Composite Reliability (CR) to assess the reliability of the questionnaire. As shown in Table 3, the Cronbach’s α values for all relevant variables in the questionnaire ranged from 0.768 to 0.881, exceeding the threshold of 0.7 (Hair, 2014). The Composite Reliability (CR) values for all variables ranged from 0.773 to 0.899, also surpassing the critical value of 0.7. In summary, the questionnaire demonstrates good reliability and satisfactory consistency.

**Table 3:** Descriptive statistics, intercorrelations, and reliability and validity measures.

Variable	Mean	Cronbach’s α	SD	AVE	Correlations					
					TC	EUI	EUR	IC	KIC	RC
TC	4.420	0.881	1.680	0.735	**0.858**					
EUI	4.566	0.859	1.438	0.703	0.186	**0.838**				
EUR	4.676	0.807	1.386	0.633	0.195	0.509	**0.796**			
IC	4.654	0.768	1.428	0.684	0.045	0.639	0.586	**0.827**		
KIC	4.624	0.862	1.441	0.707	0.315	0.600	0.616	0.615	**0.841**	
RC	4.632	0.831	1.447	0.748	0.001	0.609	0.647	0.618	0.671	**0.865**

Bold values on the diagonal are the square roots of the AVE. Values below the diagonal are correlation coefficients.

Regarding validity, this study assessed convergent validity and discriminant validity following the standard PLS-SEM procedure (Hair et al., 2017, 2019). As shown in Table 4, the factor loadings for all variables ranged from 0.776 to 0.890, exceeding the acceptable threshold of 0.7. As shown in Table 3, the Average Variance Extracted (AVE) values for all variables were higher than the acceptable threshold of 0.5 (Fornell and Larcker, 1981), indicating good convergent validity of the questionnaire.

**Table 4:** Standardized factor loadings.

Constructs	Item	Factor loading
Task complexity	TC1: I consider my tasks to be mentally demanding.	0.824
	TC2: I consider my tasks to be complex.	0.838
	TC3: I consider my tasks to require a great deal of thinking and problem-solving.	0.890
	TC4: I consider my tasks to be challenging.	0.877
Exploitative use	EUI1: I use GenAI assistants to consolidate and deepen my existing professional knowledge and skills.	0.827
	EUI2: I use GenAI assistants to continuously improve existing workflows to enhance efficiency.	0.870
	EUI3: I use GenAI assistants to select the most suitable solution from multiple alternatives for my current work.	0.831
	EUI4: I use GenAI assistants to optimize existing solutions to problems encountered in my current work.	0.825
Exploratory use	EUR1: I use GenAI assistants to explore new work ideas and possibilities.	0.807
	EUR2: I use GenAI assistants to learn new skills that are valuable for my work.	0.776
	EUR3: I use GenAI assistants to proactively explore new knowledge in certain domains.	0.811
	EUR4: I use GenAI assistants to acquire completely new knowledge that is significant for my work.	0.789
Incremental creativity	IC1: I can adapt and improve upon existing ideas in my work to address new tasks.	0.814
	IC2: I can easily modify pre-existing work procedures to fit current needs.	0.866
	IC3: I am able to utilize existing ideas or outcomes in appropriately new ways to enhance work efficiency.	0.799
Radical creativity	RC1: I propose entirely new ways of doing my work.	0.854
	RC2: I can demonstrate originality in my work.	0.883
	RC3: I can propose highly creative ideas at work.	0.856
Knowledge integration capability	KIC1: I am able to synthesize and integrate my personal expertise to solve work problems.	0.845
	KIC2: I can draw knowledge from multiple specialized fields to form new work concepts.	0.849
	KIC3: I can see clearly how different parts of my work fit together.	0.843
	KIC4: I am able to combine new work knowledge with what I already know.	0.826

Finally, three methods were employed to assess discriminant validity. First, the square roots of the AVE values for each construct were greater than the correlation coefficients between that construct and all other constructs (Fornell and Larcker, 1981). Second, we introduced the Heterotrait-Monotrait Ratio (HTMT) test. The calculated HTMT values between each variable and all others were all below the acceptable threshold of 0.85, meeting the standard for discriminant validity (Henseler et al., 2015). Third, the cross-loading method was used. The results showed that the loading of each item on its assigned construct was higher than its loadings on all other constructs, satisfying the standard for discriminant validity (Chin, 1998). In summary, the measurement scales exhibit good discriminant validity.

To address potential conceptual overlap between GenAI usage and innovation measures, we further conducted rigorous discriminant validity assessment. Results show that all HTMT values are below 0.85, and the square roots of AVE are greater than inter-construct correlations, indicating sufficient distinction between usage patterns and innovation types. Although item wording was refined to reduce semantic redundancy, future research may further develop and validate new scales to minimize conceptual proximity.

### 5.2 Hypothesis testing

This study employed SmartPLS 4 software to test the hypothesized relationships within the structural model. A Bootstrap sampling method with 5,000 resamples was applied to the 381 valid cases to conduct significance tests for the hypothesized paths. The analysis of predictive validity (*Q*^2^ test) revealed that the *Q*^2^ values for knowledge integration capability, incremental innovation, and radical innovation were 0.539, 0.473, and 0.494, respectively. As all *Q*^2^ values were greater than zero, the model demonstrates good predictive relevance. Furthermore, the analysis of R^2^ values indicates that knowledge integration capability explains 52.9% of the variance in incremental innovation and 57.7% of the variance in radical innovation. The exploitative and exploratory uses of GenAI collectively explain 57.6% of the variance in knowledge integration capability. These results demonstrate a good level of explanatory power for the overall model. Additionally, tests for the theoretical effect size (f^2^) showed that the f^2^ values for all hypothesized paths exceeded 0.02, indicating that the explanatory effects of these paths are present (Hair, 2014).

#### 5.2.1 Main effect tests

As shown in the test results in Table 5, the impact coefficient of GenAI’s exploitative use on knowledge integration is *β* = 0.326, *p* < 0.001. This indicates that exploitative use of GenAI positively influences employees’ knowledge integration capability, thus supporting H1a. The impact coefficient of GenAI’s exploratory use on knowledge integration is *β* = 0.399, *p* < 0.001. This indicates that exploratory use of GenAI positively influences employees’ knowledge integration capability, thus supporting H1b. The impact coefficient of knowledge integration capability on employees’ incremental innovation is *β* = 0.241, *p* < 0.001. This indicates that knowledge integration capability positively influences employees’ incremental innovation, thus supporting H2a. The impact coefficient of knowledge integration capability on employees’ radical innovation is *β* = 0.323, *p* < 0.001. This indicates that knowledge integration capability positively influences employees’ radical innovation, thus supporting H2b.

**Table 5:** Results of main effect tests.

Hypothesis	Path	Coefficient (β)	S.E.	*p* value	Bootstrap 95% CI	Result
H1a	EUI → KIC	0.326	0.054	0.000	[0.223, 0.432]	Support
H1b	EUR → KIC	0.399	0.052	0.000	[0.295, 0.501]	Support
H2a	KIC → IC	0.241	0.065	0.000	[0.123, 0.375]	Support
H2b	KIC → RC	0.323	0.063	0.000	[0.209, 0.458]	Support
Control path	Gender→IC	−0.009	0.075	0.908	[−0.155, 0.138]	Not Support
	Gender→RC	−0.098	0.069	0.151	[−0.233, 0.037]	Not Support
	Age→IC	0.028	0.045	0.541	[−0.060, 0.116]	Not Support
	Age→RC	−0.036	0.043	0.399	[−0.120, 0.049]	Not Support
	Education→IC	0.017	0.035	0.633	[−0.053, 0.082]	Not Support
	Education→RC	0.033	0.041	0.428	[−0.049, 0.112]	Not Support
	Job Tenure→IC	−0.067	0.052	0.196	[−0.172, 0.031]	Not Support
	Job Tenure→RC	−0.002	0.044	0.971	[−0.093, 0.081]	Not Support
	Position Level→IC	0.004	0.036	0.916	[−0.068, 0.077]	Not Support
	Position Level→RC	0.008	0.043	0.851	[−0.074, 0.095]	Not Support

#### 5.2.2 Mediating effect test

As indicated by the test results in Table 6, the indirect effects for H3a–H3d are all significant, with their 95% confidence intervals excluding zero. Specifically, exploitative use positively influences incremental creativity through knowledge integration capability (supporting H3a), and exploitative use positively influences radical creativity through knowledge integration capability (supporting H3b). Similarly, exploratory use positively influences incremental creativity through knowledge integration capability (supporting H3c), and exploratory use positively influences radical creativity through knowledge integration capability (supporting H3d).

**Table 6:** Results of mediation effect tests.

Effect	Hypothesis	Path	Coefficient (β)	*p* value	Bootstrap95%CI	Result
Indirect effect	H3a	EUI → KIC → IC	0.079	0.003	[0.038, 0.144]	Support
	H3b	EUI → KIC → RC	0.105	0.000	[0.058, 0.177]	Support
	H3c	EUR → KIC → IC	0.096	0.003	[0.045, 0.170]	Support
	H3d	EUR → KIC → RC	0.129	0.000	[0.076, 0.211]	Support
Direct effect	EUI → IC		0.367	0.000	[0.229, 0.521]	Support
	EUI → RC		0.253	0.000	[0.162, 0.361]	Support
	EUR → IC		0.251	0.000	[0.147, 0.381]	Support
	EUR → RC		0.319	0.000	[0.210, 0.447]	Support

Regarding the analysis of direct effects, the direct effects of exploitative use on both incremental and radical innovation are significant (their 95% confidence intervals exclude zero). Therefore, knowledge integration capability can be considered to play a complementary mediating role in the relationships between exploitative use and both incremental and radical innovation (Zhao et al., 2010). Similarly, the direct effects of exploratory use on both incremental and radical innovation are significant, indicating that knowledge integration capability also serves as a complementary mediator in the relationships between exploratory use and both types of innovation.

Furthermore, we employed a bootstrap-based test of path coefficient differences (Rodríguez-Entrena et al., 2018) to examine whether the effects of exploitative and exploratory GenAI use on distinct innovation types differed significantly. The results showed that the effect of exploitative use on incremental innovation was significantly stronger than its effect on radical innovation (Δ*β* = 0.114, *t* = 2.128, *p* < 0.05). Similarly, the effect of exploratory use on radical innovation was significantly stronger than its effect on incremental innovation (Δ*β* = 0.068, *t* = 2.086, *p* < 0.05).

#### 5.2.3 Moderating effect test

To further examine whether task complexity significantly moderates the relationship between GenAI usage and knowledge integration, an analysis of the moderating effect was conducted. The specific results are presented in Table 7. The results indicate that task complexity positively moderates the effect of exploitative GenAI use on knowledge integration capability (*β* = 0.131, *p* < 0.05), thus supporting H4a. Similarly, task complexity positively moderates the effect of exploratory GenAI use on knowledge integration capability (*β* = 0.159, *p* < 0.05), supporting H4b.

**Table 7:** Results of moderating effect tests.

Hypothesis	Path	Coefficient (*β*)	S.E.	*p* value	Bootstrap 95% CI	Result
H4a	TC × EUI → KIC	0.131	0.059	0.028	[0.011, 0.245]	Support
H4b	TC × EUR → KIC	0.159	0.062	0.011	[0.033, 0.274]	Support

**Figure 2:**
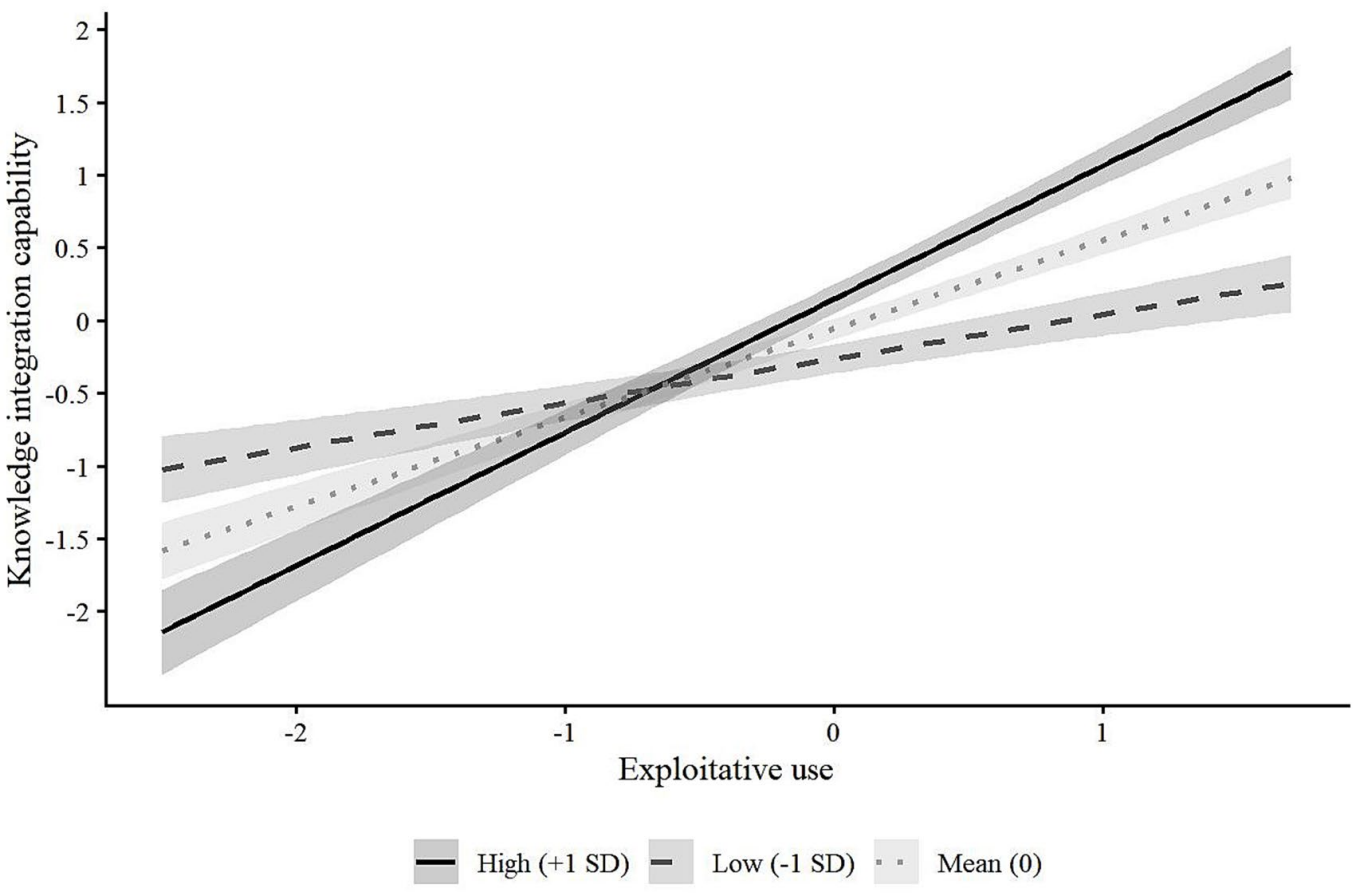
The moderating effect of task complexity on the relationship between exploitative use and knowledge integration capability.

**Figure 3:**
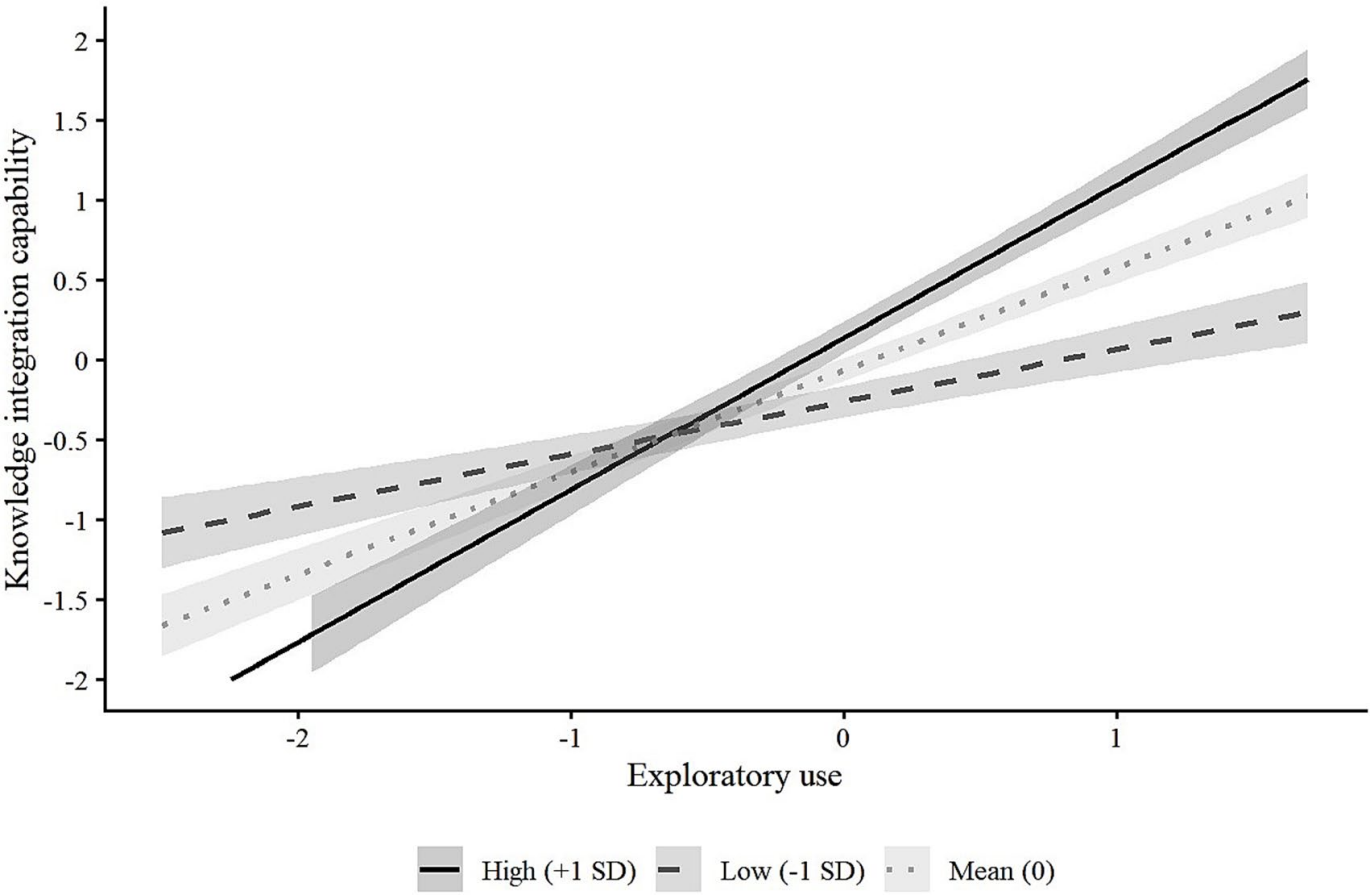
The moderating effect of task complexity on the relationship between exploratory use and knowledge integration capability.

To provide a clearer interpretation of the moderating effects, this study followed McCabe et al. (2018) to visualize the interactions (Figures 2, 3). Predicted values of knowledge integration were plotted across the full range of GenAI use at low (−1 SD), mean, and high (+1 SD) levels of task complexity, along with 95% confidence intervals. As shown, the slopes for both exploitative and exploratory use increase with task complexity, indicating that their positive effects on knowledge integration capability are stronger under higher task complexity. The separation of confidence bands further supports the robustness of these moderating effects.

Overall, task complexity positively moderates the impact of two usage modes of GenAI on knowledge integration capability. The higher the task complexity, the stronger the promotional effect of GenAI usage on knowledge integration capability.

## 6 Conclusions and implications

### 6.1 Research conclusions

This study constructs a moderated mediation model to empirically examine how exploitative and exploratory GenAI usage patterns shape employees’ incremental and radical innovative behavior, with knowledge integration capability as the mediating mechanism and task complexity as the contextual moderator. Based on the analysis of 381 valid multi-wave questionnaire data from knowledge-intensive enterprises in China, the following four core conclusions are drawn (aligning with the theoretical framework and empirical findings):

First, exploitative and exploratory GenAI usage patterns exert differential effects on distinct types of innovative behavior. Exploitative GenAI use—characterized by refining existing workflows, optimizing known solutions, and deepening domain-specific expertise—exerts a significantly stronger positive effect on incremental innovation than on radical innovation (Δ*β* = 0.114, *t* = 2.128, *p* < 0.05). In contrast, exploratory GenAI use—defined by open-ended inquiry, cross-domain knowledge acquisition, and experimental idea generation—demonstrates a more pronounced positive impact on radical innovation compared with incremental innovation (Δβ = 0.068, *t* = 2.086, *p* < 0.05). This confirms that the value of GenAI for innovation lies not in mere usage, but in the alignment between usage patterns and innovation objectives.

Second, employees’ knowledge integration capability serves as a critical sequential mediator linking GenAI usage patterns to innovative outcomes. Both exploitative (*β* = 0.326, *p* < 0.001) and exploratory (*β* = 0.399, *p* < 0.001) GenAI use positively enhance employees’ ability to synthesize heterogeneous knowledge, which in turn facilitates incremental (*β* = 0.241, *p* < 0.001) and radical (*β* = 0.323, *p* < 0.001) innovation. Specifically, knowledge integration transmits 21.5% of the effect of exploitative use on incremental innovation (0.079/0.367) and 41.5% of the effect of exploratory use on radical innovation (0.129/0.319), highlighting it as the core cognitive conduit through which GenAI transforms into innovative performance.

Third, task complexity positively moderates the relationship between GenAI usage patterns and knowledge integration capability. For both exploitative (*β* = 0.131, *p* < 0.05) and exploratory (*β* = 0.159, *p* < 0.05) GenAI use, the positive effect on knowledge integration is significantly stronger under high task complexity (M + 1SD) than under low task complexity (M-1SD). This is because high-complexity tasks (marked by goal ambiguity and information overload), making GenAI’s cognitive augmentation more salient and its outputs more conducive to integrative thinking.

Fourth, the mediating role of knowledge integration is complementary rather than exclusive. The direct effects of both GenAI usage patterns on innovative behavior remain significant after controlling for knowledge integration, indicating that GenAI influences innovation through dual pathways: one via cognitive integration of AI-generated knowledge, and the other via direct motivational stimulation (e.g., competence satisfaction from efficient task completion, autonomy fulfillment from exploratory learning). This dual-path mechanism explains the “innovation paradox” in prior research and enriches the understanding of human-AI co-creativity.

### 6.2 Theoretical contributions

From the perspective of employee management and organizational innovation, this study makes three distinct theoretical contributions to the literature on GenAI and employee innovation:

First, it advances the conceptualization of GenAI usage behavior in organizational contexts. Moving beyond the unidimensional “adoption/usage frequency” perspective prevalent in existing research (Budhwar et al., 2023; Noy and Zhang, 2023), this study theoretically grounds and empirically validates a dual-dimensional taxonomy of GenAI usage (exploitative vs. exploratory) based on cognitive processing and knowledge goals. This reframing complements existing research and addresses the gap of “how GenAI use shapes innovation outcomes.

Second, it demystifies the “black box” between GenAI and differentiated innovation outcomes. Prior studies have generally confirmed GenAI’s positive effect on creativity (Shao et al., 2022; Yin et al., 2024) but failed to explain why it sometimes fosters incremental adjustments and sometimes drives radical transformations—especially in marketing contexts such as content creation, customer relationship management, and market expansion. This study identifies knowledge integration capability as the core mediating mechanism and clarifies dual pathways: exploitative use fuels incremental innovation (e.g., optimizing ad copy, refining customer segmentation models) through efficiency-oriented knowledge refinement, while exploratory use catalyzes radical innovation (e.g., developing novel customer engagement models, creating disruptive brand narratives) through boundary-expanding knowledge recombination. This completes the proposed theoretical sequence of “GenAI usage pattern→knowledge integration→innovation type,” enhancing the theoretical precision of human-AI co-creation research.

Third, it integrates exploration–exploitation framework with knowledge integration theory in AI-driven innovation and identifies task complexity as a key boundary condition (Bo et al., 2023). This study provides a cognitive-knowledge perspective to explain human-AI co-creativity, which complements motivational perspectives in prior research (Zhang et al., 2025), particularly where employees often face dual demands of routine optimization and creative breakthroughs. It addresses the practical question of “when GenAI is most effective for employee innovation.” For example, in high-complexity marketing tasks (e.g., cross-border market entry, integrated communication planning), GenAI’s value is more pronounced, which provides a theoretical basis for targeted AI deployment.

GenAI may generate homogenized outputs and reduce creative diversity, while only autonomous exploratory use can effectively promote radical innovation. This study provides correlational and predictive evidence rather than strict causal conclusions.

### 6.3 Managerial implications

Based on the empirical findings, this study offers four evidence-based recommendations for managers to leverage GenAI to enhance organizational innovation capacity:

First, align GenAI usage strategies with innovation goals and task characteristics. For incremental objectives—such as optimizing existing campaign workflows, improving customer service response efficiency, or refining data analysis models—organizations should scaffold exploitative GenAI use. Specifically, marketing departments can: (1) develop industry-specific GenAI templates (e.g., standardized ad copy generation, customer feedback analysis frameworks); (2) build a prompt library for routine marketing tasks (e.g., social media content drafting, market research report outlines); (3) fine-tune GenAI models based on the company’s historical marketing data, brand voice, and customer profiles to ensure output consistency and relevance. For radical goals—such as launching new products, entering untapped markets, or developing innovative customer engagement strategies—managers should incentivize exploratory GenAI use, such as organizing “marketing innovation sprints” (e.g., using GenAI to explore emerging consumer trends), hosting cross-functional ideation forums (e.g., collaborating with R&D and sales teams to generate disruptive marketing concepts via GenAI), and providing access to diverse knowledge corpora (e.g., industry whitepapers, global consumer behavior data) to stimulate cross-domain thinking.

Second, systematically cultivate employees’ knowledge integration capability. As the core cognitive link between GenAI inputs and employee innovation, this capability requires targeted development. Managers can: (1) implement structured training on metacognitive strategies tailored to practical scenarios, such as teaching employees to compare GenAI-generated content with brand positioning, detect logical inconsistencies in AI-driven market insights, and use analogical mapping to connect AI outputs with customer needs; (2) embed integration practices into daily workflows, such as requiring post-task reflections (e.g., “How did you integrate GenAI’s suggestions with your existing marketing knowledge?”) for AI-assisted projects like campaign planning or customer journey mapping; (3) foster communities of practice where employees share experiences of integrating GenAI into their work (e.g., successful cases of using AI to combine customer data with creative concepts) and collectively validate AI-generated insights to avoid blind reliance.

Third, prioritize GenAI deployment in high-complexity domains. Given task complexity’s moderating role, firms should focus GenAI resources on high-value, high-complexity tasks where its cognitive augmentation effect is most significant. Examples include: (1) strategic foresight (e.g., using GenAI to analyze macroeconomic trends, competitor strategies, and consumer behavior changes to predict market opportunities); (2) regulatory compliance navigation (e.g., leveraging GenAI to ensure marketing content complies with regional laws and cultural norms in cross-border campaigns); (3) interdisciplinary R&D collaboration (e.g., using GenAI to integrate technical product features with customer pain points for innovative value propositions); (4) large-scale customer segmentation and personalized marketing (e.g., analyzing multi-source customer data to develop tailored communication strategies). For routine, low-complexity tasks (e.g., basic data entry, simple social media posts), GenAI deployment can be limited to avoid unnecessary resource waste and cognitive dependency.

Fourth, institutionalize human-AI co-creativity through aligned incentives and culture. Managers should: (1) design recognition systems that reward effective GenAI integration rather than mere usage—for example, establishing awards such as “Best AI-Augmented Marketing Campaign,” “Most Insightful GenAI-Driven Market Analysis,” or “Innovative Use of AI in Customer Engagement” to encourage employees to combine AI outputs with their professional judgment; (2) cultivate leadership narratives that emphasize “intelligence augmentation” over automation, highlighting that GenAI is a tool to enhance employee creativity rather than replace human expertise (e.g., showcasing cases where AI-generated ideas were refined by marketers to better align with brand values); (3) embed psychological safety mechanisms to normalize experimentation—such as conducting blameless post-mortems of AI-assisted projects (focusing on what can be learned rather than assigning blame for failures) and allowing employees to test unconventional GenAI applications in low-risk scenarios (e.g., pilot campaigns for niche audiences). These measures will help transform GenAI from a transactional tool into a strategic enabler of ambidextrous innovation, simultaneously strengthening adaptive efficiency and transformative breakthrough capacity.

### 6.4 Research limitations and future directions

This study has several limitations that point to promising avenues for future research:

First, this study relies on single-source self-reported data. Future research may adopt supervisor-rated innovation and objective GenAI usage logs. Experimental designs (e.g., randomly assigning work teams to exploitative vs. exploratory GenAI use groups) can also be used to verify the causal effects of usage patterns on innovation.

Second, although we refined item wording to reduce semantic overlap, some items still show conceptual proximity. Future studies may develop new scales with greater discriminant validity.

Third, although this study captures the broad orientations of GenAI exploitative and exploratory use, it does not reflect fine-grained, context-specific behavioral details in workplace practice. The current measurement focuses on general usage tendencies rather than concrete interaction processes, such as prompt design, output evaluation, and knowledge integration depth. Future research could develop and validate more fine-grained behavioral scales to capture nuanced GenAI application behaviors, and use objective interaction logs or natural language processing to record real-world GenAI engagement patterns.

Fourth, this study is limited to knowledge-intensive enterprises in China, which may restrict the generalizability of its findings. The sample does not cover traditional industries, low-cognitive-intensity sectors, or cross-national contexts, where GenAI usage motivations and innovation outcomes may differ. Future studies can extend the sampling framework to diverse industries and cultural backgrounds to test the cross-context robustness of the theoretical model, and further compare the differential effects of GenAI usage across industry and regional boundaries.

## Data Availability

The original contributions presented in the study are included in the article/supplementary material, further inquiries can be directed to the corresponding author.

## Footnotes

1 Generative Artificial Intelligence Application Development Report (2025).
